# Clinical efficacy and patient outcomes following the application of minimally invasive tooth extraction techniques in the surgical removal of maxillary third molars

**DOI:** 10.1097/MD.0000000000048889

**Published:** 2026-05-29

**Authors:** Yanpei Wei

**Affiliations:** a Tangshan People’s Hospital, Tangshan, Hebei Province, China.

**Keywords:** clinical application, maxillary third molar, minimally invasive tooth extraction technology, prognostic study

## Abstract

This study aimed to analyze the clinical application and prognosis of minimally invasive tooth extraction techniques for maxillary third molars. A total of 110 patients with impacted maxillary third molars were included and retrospectively analyzed. Fifty-three patients in the control group were treated with traditional chisel and crown extraction, and 57 patients in the observation group were treated with minimally invasive extraction. The operative effect, perioperative symptoms, and postoperative complications were compared between the 2 groups. The operation time and alveolar fossa healing time in the observation group were (16.30 ± 3.56) minutes and (7.80 ± 2.59) days shorter than those in the traditional tooth extraction group, and the blood loss was (6.78 ± 3.01) mL less than that in the control group. The alveolar bone absorption was better in 36 cases of type I, 16 cases of type II, 4 cases of type III, and 1 case of type IV. The Visual Analog Scale score and wound swelling score in the observation group were lower than those in the traditional tooth extraction group, and the degree of mouth opening limitation was 0 in 32 cases, 1 in 19 cases, 2 in 3 cases, and 3 in 3 cases, and the results were better than those in the control group. There were 3 cases of adjacent teeth injury, 0 cases of root fracture, 3 cases of infection, and 5 cases of lip numbness in the minimally invasive tooth extraction group, and the number of postoperative complications was less than that in the control group, with statistical significance (*P* < .05). Minimally invasive tooth extraction in the extraction of impacted maxillary third molar has better surgical effect, less perioperative symptoms and lower postoperative complications, which can improve the efficiency and safety of the operation and has certain clinical value and application prospects in modern stomatology.

## 1. Introduction

As the human jaw has evolved and regressed to varying degrees, the eruption space for the maxillary third molars has become increasingly restricted. This trend has resulted in a significant reduction in both the length and width of the maxilla, hindering the normal growth of third molars and making them more susceptible to various forms of impaction.^[1]^ Impacted wisdom teeth not only fail to emerge properly into the occlusal plane but can also become embedded in the jawbone or under the gums at various angles, potentially causing a range of oral health problems.

Clinically, the most common complication of impacted wisdom teeth is pericoronitis, which occurs when partially erupted wisdom teeth form a pocket between the crown and gum, trapping food debris and bacteria and leading to infection. In addition, neighboring teeth can develop caries due to difficulty in cleaning, which can progress to pulpitis and periodontitis, posing a serious threat to oral health.^[2]^ More critically, if the infection is not controlled in time, it can spread to the surrounding soft tissues, causing interdental infections or even to the jawbone, leading to osteomyelitis, which can severely affect the patient’s daily life and work. In addition, impacted wisdom teeth can adversely affect the alignment of the maxillary anterior teeth, causing crowding and displacement and potentially disrupting the normal occlusal relationship, leading to occlusal dysfunction. In a retrospective study conducted in Iran, 57.4% of patients attending dental radiology clinics had at least 1 tooth affected by the third molar.^[3]^ Long-term occlusal imbalance can further trigger temporomandibular joint (TMJ) disorders such as TMJ dysfunction syndrome, characterized by limited mouth opening, joint pain, and clicking sounds, which can significantly affect chewing function and facial comfort.

Given the numerous potential risks associated with impacted wisdom teeth, the field of dentistry has placed great emphasis on their early detection and management. The latest clinical guidelines clearly recommend the timely extraction of pathologically erupted third molars – those that are impacted and either cause or are likely to cause the aforementioned complications.^[4]^ This recommendation aims to interrupt the progression of the disease through preventive extraction, thereby protecting the oral health of patients and improving their quality of life.^[5]^

In the treatment of maxillary third molar extractions, traditional tooth extraction techniques often involve the use of chisels and splitting to remove impacted teeth. However, these methods are limited by the restricted surgical field, which results in poor visibility of the affected area.^[6,7]^ As a result, the ability to control the angle and force during the procedure is reduced, increasing the risk of complications such as retained root fragments and damage to adjacent teeth.^[8,9]^ In addition, the forces exerted in various directions on the impacted tooth may prolong the duration of the procedure and increase the likelihood of postoperative infection.^[10,11]^ In contrast, minimally invasive extraction techniques offer advantages such as smaller wound size, reduced postoperative pain, and faster healing. Minimally invasive extraction involves the use of a specialized micropowered elongated high-speed turbine drill to section the tooth crown after anesthesia, followed by the insertion of minimally invasive extraction instruments between the periodontal membrane and the alveolar bone to separate them,^[12]^ allowing complete loosening and removal of the tooth without damaging the surrounding tissues. A prospective clinical observational study demonstrated^[5]^ that minimally invasive extraction has a higher success rate and significantly reduces the need for flap surgery compared with conventional extraction techniques, highlighting its advantages in the removal of impacted teeth. Another observational study^[13]^ found that the success rate of minimally invasive extraction is related to the force applied and the type of tooth growth. However, the results of minimally invasive extraction of maxillary third molars have not been investigated in the existing studies. Therefore, in this study, 110 patients with impacted maxillary third molars who underwent extraction in the Department of Dentistry from June 2022 to May 2023 were selected to compare the clinical applications and outcomes of minimally invasive extraction and conventional extraction methods.

## 2. Data and methods

### 2.1. General information

This study was approved by the Ethics Committee of Tangshan People’s Hospital (Approval No. TSPH-2022-061). A total of 110 cases of impacted maxillary third molar extractions were retrospectively collected and divided into 2 groups according to the extraction method. In the traditional tooth extraction group, 53 patients underwent conventional extraction, including 27 males and 26 females, aged 19 to 51 years (mean ± standard deviation (SD): 38.56 ± 3.78), with 25 impacted teeth on the left and 28 on the right. In the minimally invasive extraction group, 57 patients underwent minimally invasive extraction, including 29 males and 28 females, aged 19 to 53 years (mean ± SD: 39.70 ± 3.85), with 30 impacted teeth on the left and 27 on the right. There were no significant differences in sex, age, or tooth position between the 2 groups (*P* > .05).

Inclusion criteria were as follows: all patients were diagnosed with impacted maxillary third molars according to Clinical Stomatology; jaw and crown resistance of adjacent teeth; complete clinical data; and agreed to the tooth extraction plan, knew about the research (approved by the Ethics Committee), and signed the consent form. Exclusion criteria were as follows: severe periodontitis or periapical periodontitis; maxillofacial malignant tumors; pregnancy, lactation, and menstruation; and serious systemic diseases or mental diseases.

### 2.2. Methods

#### 2.2.1. Preoperative preparation

The patients in both groups were examined by preoperative imaging, and oral X-ray curved sectional films were taken to determine the position and specific shape of impacted teeth, to clarify the actual anatomical relationship between the root tip of the affected tooth and the adjacent teeth, and to make a surgical plan. Local anesthesia was performed on the operation area; first, standard disinfection was carried out, and disinfection towels were laid, then block anesthesia or local infiltration anesthesia was used to anesthetize the lips, and at the same time, nerve block anesthesia or local infiltration anesthesia was used to anesthetize the palate.

#### 2.2.2. Traditional tooth extraction

When performing tooth extraction, the gingiva is cut by making a triangular flap incision to ensure that the crown is fully exposed. Then, the bone covering the buccal side and maxillofacial region is removed with a chisel, and then the crown is split, and the part obstructing the crown and root of the adjacent tooth is removed, so as to extract the crown and root. During the operation, all the residues in the alveolar fossa, including teeth, granulation tissue, and bone debris, should be carefully removed, and the alveolar fossa should be washed and disinfected with normal saline to ensure fresh blood oozing out. Subsequently, the periosteal flap was reset and sutured, and measures were taken to stop bleeding. After the operation, the patient needs to bite with gauze to stop bleeding, and carry out a 24-hour ice compress in the operating area to reduce swelling and pain. Avoid gargling within 24 hours after the operation, and keep a light diet for the next 3 to 5 days, while paying attention to oral hygiene. In addition, antibiotics should be taken orally for 3 days after the operation to prevent infection.

#### 2.2.3. Minimally invasive tooth extraction

During tooth extraction, a triangular flap incision method is used to open the gums to ensure that the crown is completely exposed. Then, a high-speed turbine machine with an inclination angle of 45 degrees was used to remove the bone tissue on the surface of the crown until the maximum circumference of the crown was exposed. Subsequently, the crown and root were separated to eliminate the obstruction of adjacent teeth, and the periodontal ligament was cut off by a minimally invasive technique to separate the root from the alveolar bone. Pull out the impacted teeth with slight rotating force, and thoroughly clean the alveolar fossa to ensure that there is no tooth, granulation tissue, or bone debris left. Rinse the alveolar fossa with normal saline and ensure that fresh blood flows out. Subsequently, the periosteal flap was reset and sutured, and pressure was applied to stop bleeding.^[14]^ After the operation, the patient was instructed to use gauze for occlusal hemostasis, and a 24-hour ice compress was performed in the operative area to reduce swelling and pain. Avoid gargling within 24 hours after the operation, maintain a light diet for 3 to 5 days, and pay attention to oral hygiene. In addition, patients should take antibiotics orally for 3 days after the operation to prevent infection.

To minimize operator-related bias, all extractions in both the control and observation groups were performed by the same senior oral surgeon with more than 10 years of clinical experience in oral and maxillofacial surgery. This ensured consistency in surgical technique and judgment across both traditional and minimally invasive extraction procedures.

### 2.3. Observation indicators

#### 2.3.1. Evaluation index of surgical effect

The evaluation indices of the operation effect were compared between the 2 groups, including operation time, alveolar fossa healing time, alveolar bone absorption degree, and blood loss. Operation time reflects the complexity and efficiency of tooth extraction operation, and long-term operation may increase the risk of intraoperative complications, such as excessive bleeding or tissue damage. The healing time of the alveolar fossa reflects the recovery speed of patients, and rapid healing usually means good prognosis and less risk of infection. The integrity of the alveolar bone is a method used to evaluate the health status of the alveolar bone. This scoring system is helpful to identify the degree of alveolar bone loss, bone status, and the possibility and complexity of reconstruction. The amount of bleeding can evaluate the fineness of the surgical operation and the effectiveness of hemostasis measures.

#### 2.3.2. Perioperative symptom evaluation index

The perioperative symptoms of the 2 groups were compared, including the degree of pain, the degree of wound swelling, and the degree of mouth opening limitation. Visual Analog Scale (VAS)^[15]^ was used to evaluate the degree of pain, where 0 was painless, 1 to 3 was mild pain, 4 to 7 was moderate pain, and 8 to 10 was severe pain. The evaluation criteria for wound swelling^[16]^ are as follows: a score of 0 indicates that the patient has no swelling; a score of 1, and the swelling is limited to the upper lip area; when the score is 2, the swelling covers not only the upper lip, but also the disappearance of the nasolabial groove; a score of 3 indicates that the swelling has spread to the upper lip and lower eyelid, and the nasolabial groove has disappeared. The degree of mouth opening limitation can be quantified by a mouth opening limitation score, and the size of mouth opening can be determined by a measuring tool oral caliper, to evaluate the degree of mouth opening limitation. Zero degrees means that the mouth opening distance is greater than or equal to 25 mm, 1 degree means that the mouth opening distance is between 20 and 25 mm, 2 degrees means that the mouth opening distance is between 10 and 20 mm, and 3 degrees means that the mouth opening distance is <10 mm.

#### 2.3.3. Evaluation index of postoperative complications

Seven days after operation, the complications of the 2 groups were collected, including adjacent tooth injury, maxillary sinus or nasal communication, root tearing, infection, dry socket, and lip numbness.

### 2.4. Statistical methods

SPSS version 23.0 (IBM Corp., Armonk) statistical software was used to analyze the data, and the counting data was expressed as a percentage; the χ^2^ test was used. If the measurement data conforms to the normal distribution, it is expressed as mean ± SD (*x̄* ± *s*), and *t* test was adopted; non-normal distribution or uneven variance is represented by the median, and the difference is statistically significant with *P* < .05.

### 2.5. Post hoc power analysis

To further validate the adequacy of the sample size and the reliability of the statistical conclusions, a post hoc power analysis was performed using G*Power version 3.1 software (Heinrich-Heine-Universität Düsseldorf, Düsseldorf, Germany). Based on the observed differences in operative time between the 2 groups (mean difference ≈ 12.5 minutes, SD ≈ 4.5), the calculated effect size (Cohen d) was approximately 2.78. Given the total sample size of 110 (53 in the control group and 57 in the observation group), the post hoc statistical power (1−β) exceeded 0.95 at a significance level of α = 0.05. Similarly, for perioperative VAS pain scores (effect size d ≈ 0.8), the power was approximately 0.88. These results indicate that the present study had sufficient statistical power to detect clinically meaningful differences between the 2 groups.

## 3. Results

### 3.1. Comparison of surgical effect evaluation

The results of the comparison between the 2 groups showed that the operation time and alveolar fossa healing time of the minimally invasive tooth extraction group were (16.30 ± 3.56) minutes and (7.80 ± 2.59) days shorter than those of the traditional tooth extraction group, and the blood loss was (6.78 ± 3.01) mL less than that of the control group. The alveolar bone absorption was better in 36 cases of type I, 16 cases of type II, 4 cases of type III, and 1 case of type IV (Table 1).

**Table 1:** Evaluation and comparison of surgical effect between 2 groups of patients.

Group	Operation time (min)	Alveolar fossa healing time (d)	Degree of alveolar bone absorption (n, %)				Blood loss (mL)
			Type I	Type II	Type III	Type IV	
Control group (n = 53)	28.84 ± 4.86	17.48 ± 2.60	19 (35.85)	21 (39.62)	9 (16.98)	4 (7.55)	10.44 ± 3.07
Observation group (n = 57)	16.30 ± 3.56	7.80 ± 2.59	36 (63.16)	16 (28.07)	4 (7.02)	1 (1.75)	6.78 ± 3.01
*T*/χ^2^ value	15.043	62.422	9.520				6.861
*P* value	<.001	<.001	.043				<.001

### 3.2. Comparison of perioperative symptom evaluation

The comparison results between the 2 groups showed that the VAS score and wound swelling score in the minimally invasive tooth extraction group were lower than those in the traditional tooth extraction group, and mouth opening was limited in 32 cases of 0 degrees, 19 cases of 1 degree, 3 cases of 2 degrees, and 3 cases of 3 degrees, and the results were better than those in the control group (*P* < .05; Table 2).

**Table 2:** Comparison of perioperative symptoms between the 2 groups.

Group	VAS score	Degree of wound swelling	Degree of mouth opening restriction (n, %)			
			0 degrees	1 degrees	2 degrees	3 degrees
Control group (n = 53)	5.66 ± 1.88	2.09 ± 1.33	21 (39.62)	15 (28.30)	9 (16.98)	8 (15.09)
Observation group (n = 57)	5.66 ± 1.88	1.47 ± 1.14	32 (56.14)	19 (33.33)	3 (5.26)	3 (5.26)
*T*/χ^2^ value	13.803	2.475	7.8919			
*P* value	<.001	.017	.045			

VAS = Visual Analog Scale.

### 3.3. Evaluation and comparison of postoperative complications

The comparison between the 2 groups showed that after tooth extraction, there were 3 cases of adjacent tooth injury, 0 cases of root fracture, 3 cases of infection, and 5 cases of lip numbness in the minimally invasive tooth extraction group, and the number of complications was lower than that in the control group (*P* < .05; Table 3).

**Table 3:** Comparison of postoperative complications between the 2 groups (n, %).

Group	Adjacent tooth injury	Maxillary sinus or nasal cavity communication	Root tear	Infect	Dry socket	Lip numbness
Control group (n = 53)	10 (18.87)	4 (7.55)	8 (15.09)	11 (20.75)	7 (13.21)	14 (26.42)
Observation group (n = 57)	3 (5.26)	0 (0.00)	1 (1.75)	3 (5.26)	1 (1.75)	5 (8.77)
χ^2^	4.878	3.860	5.395	4.599	4.400	4.398
*P* value	.038	.119	.033	.044	.062	.045

## 4. Discussion

### 4.1. Minimally invasive tooth extraction is more effective in the extraction of impacted teeth of the maxillary third molar

The impacted maxillary third molar, usually called the wisdom tooth, may cause many problems, such as pain, infection, or damage to adjacent teeth. Although it is necessary to remove these impacted wisdom teeth, they are located at the last end of the mouth, which makes the operation more difficult, and sometimes they are partially hidden under the gums or completely buried in the bones, which further increases the complexity of tooth extraction.^[17]^ Moreover, the maxillary third molar is close to important anatomical structures, such as the maxillary sinus and buccal nerve, so care should be taken when extracting teeth to avoid damaging these structures.^[18]^ In this case, the traditional tooth extraction surgery often requires a wide range of incisions and bone tissue removal, which not only damages the surrounding soft and hard tissues but also may affect the health of adjacent teeth. In contrast, minimally invasive tooth extraction only operates in the minimum necessary range by using fine instruments and techniques,^[19]^ such as high-precision drills and minimally invasive tooth extraction instruments, thus significantly reducing tissue damage.^[5]^ In this study, the minimally invasive tooth extraction group had a shorter operation time and less intraoperative bleeding, which shows that minimally invasive tooth extraction technology can reduce the operation time and is highly efficient. This may be because minimally invasive surgery usually uses fine surgical instruments and magnifying visual equipment, which can help doctors operate accurately and reduce unnecessary cutting and drilling, thus shortening the operation time, which is consistent with the research of Nie et al^[20]^ and other scholars.

At the same time, this study also found that the healing time of the alveolar fossa in the minimally invasive tooth extraction group was shorter, and the absorption degree of alveolar bone was higher in type I and type II. Also, due to the advantages of precise and rapid minimally invasive tooth extraction, the damage to surrounding teeth and soft and hard tissues was reduced, which helped to heal the alveolar fossa faster.

### 4.2. Minimally invasive extraction of impacted maxillary third molars exhibits mild perioperative symptoms

In traditional tooth extraction procedures, larger incisions and more extensive tissue manipulation are often required, which frequently results in greater postoperative pain and swelling. These symptoms are typically caused by significant trauma to the surrounding soft tissues and alveolar bone. Minimally invasive extraction techniques, on the other hand, allow for more precise removal of the impacted teeth, minimizing unnecessary damage to the surrounding tissues. By preserving the integrity of the alveolar bone and gingival tissue, these techniques significantly reduce the local inflammatory response, which in turn alleviates postoperative pain and swelling.

One of the key advantages of minimally invasive extraction is the reduced perioperative trauma. Traditional techniques often involve more forceful manipulation and longer incisions, leading to increased soft tissue disruption and subsequent inflammation. In contrast, the minimally invasive approach focuses on using specialized instruments, such as high-speed micro drills, to section the tooth into smaller pieces, allowing for controlled and less traumatic removal. This method reduces the extent of tissue damage, helping to decrease the overall inflammatory response and promote faster healing. As a result, patients experience less postoperative discomfort, including swelling, pain, and limited jaw mobility.

Moreover, this study found that patients undergoing minimally invasive extraction experienced a lower degree of mouth opening limitation postoperatively. In traditional extraction procedures, patients are often required to open their mouths wider for longer periods to provide adequate visibility and access to the impacted tooth. This prolonged and excessive strain on the TMJ and masseter muscle can lead to postoperative discomfort, including muscle fatigue, joint pain, and restricted mouth opening. Minimally invasive extraction, with its focus on reducing surgical trauma and preserving surrounding tissues, mitigates this issue by requiring less jaw extension and decreasing the strain on the TMJ. This contributes to a more comfortable postoperative recovery, with less muscle soreness and reduced risk of TMJ dysfunction.

The findings of this study are consistent with previous research that has demonstrated the advantages of minimally invasive techniques in reducing postoperative symptoms such as pain, swelling, and mouth opening limitation.^[13]^ By minimizing surgical trauma and optimizing patient outcomes, minimally invasive extraction represents a superior alternative to traditional tooth extraction methods, particularly for impacted maxillary third molars. The reduced postoperative discomfort and faster recovery associated with minimally invasive techniques underscore their growing importance in modern dental surgery.

### 4.3. Minimally invasive tooth extraction and its low complication rate after impacted maxillary third molar removal

The complication rate following the extraction of impacted maxillary third molars using minimally invasive techniques is notably low. This is largely due to the precise nature of the procedure, which involves smaller incisions and minimizes unnecessary manipulation of surrounding tissues. By reducing the extent of trauma to the alveolar bone and soft tissues, minimally invasive tooth extraction shortens the operative time and decreases the likelihood of common postoperative complications.

Minimally invasive tooth extraction is designed to cause the least possible disruption to surrounding anatomical structures. Traditional extraction methods often require extensive incisions and tissue retraction, increasing the risk of complications such as infection, adjacent tooth injury, root fractures, and nerve damage. In contrast, the minimally invasive approach employs advanced tools and techniques to carefully section and remove the tooth with minimal force and trauma. This controlled precision not only preserves the integrity of the surrounding tissues but also reduces postoperative complications, enhancing patient outcomes.

In this study, the complication rates for patients in the minimally invasive extraction group were significantly lower compared with those in the control group. Specifically, only 3 cases of adjacent tooth injury, 0 cases of root fractures, 3 cases of postoperative infection, and 5 cases of lip numbness were observed in the minimally invasive group. These findings are particularly important, as adjacent tooth injury and root fracture are common concerns during third molar extraction due to the proximity of the impacted tooth to surrounding teeth and roots. The absence of root fractures in the minimally invasive group highlights the effectiveness of this technique in preserving tooth integrity and reducing trauma during the procedure.

Additionally, the reduced rate of infection observed in the minimally invasive group further underscores the benefits of this technique. Postoperative infections are often linked to prolonged surgical time, excessive tissue manipulation, and larger wound sites – factors that are minimized in minimally invasive procedures. The smaller incisions and shorter operative duration limit the exposure of the surgical site to bacterial contamination, leading to a lower risk of infection and faster wound healing.

### 4.4. Clinical generalizability and limitations

The present study demonstrates that minimally invasive tooth extraction provides distinct advantages in reducing operative time, intraoperative bleeding, and postoperative complications. These findings highlight the potential for wider clinical application of this technique, particularly in dental and oral surgery settings where patient comfort, healing speed, and surgical precision are essential. Due to its minimally traumatic nature, this approach is especially suitable for elderly patients, individuals with systemic diseases, and those with higher aesthetic or functional requirements. Moreover, the required instruments and surgical protocols can be readily implemented in general dental practice following appropriate professional training, supporting its clinical generalizability.

However, several limitations should be recognized. First, this study was retrospective and conducted in a single institution with a limited sample size, which may restrict the extrapolation of the findings to broader populations. Second, all extractions were performed by 1 experienced surgeon, ensuring procedural consistency but limiting generalization to practitioners with different experience levels. Finally, the short follow-up period precluded evaluation of long-term outcomes such as alveolar bone remodeling and masticatory function recovery. Future multicenter, prospective studies with larger cohorts and extended follow-up are warranted to validate these findings and further assess their long-term clinical implications.

## 5. Conclusion

Minimally invasive tooth extraction significantly improves surgical efficiency and safety compared with traditional extraction techniques. By reducing incision size, operative time, and surgical trauma, it offers patients a less painful and faster recovery experience while minimizing postoperative complications. These advantages support its growing adoption in clinical dentistry as a reliable and patient-centered approach.

In summary, minimally invasive extraction represents a valuable advancement in modern oral surgery, providing enhanced outcomes and expanding the prospects for safer and more efficient management of impacted maxillary third molars.

## Author contributions

**Conceptualization:** Yanpei Wei.

**Data curation:** Yanpei Wei.

**Formal analysis:** Yanpei Wei.

**Funding acquisition:** Yanpei Wei.

**Investigation:** Yanpei Wei.

**Writing – original draft:** Yanpei Wei.

**Writing – review & editing:** Yanpei Wei.
